# On-Board, Real-Time Preprocessing System for Optical Remote-Sensing Imagery

**DOI:** 10.3390/s18051328

**Published:** 2018-04-25

**Authors:** Baogui Qi, Hao Shi, Yin Zhuang, He Chen, Liang Chen

**Affiliations:** 1 Beijing Key Laboratory of Embedded Real-Time Information Processing Technology, Beijing Institute of Technology, Beijing 100081, China; qibaogui@bit.edu.cn (B.Q.); zhuangyin640829@163.com (Y.Z.); chenhe@bit.edu.cn (H.C.); chenl@bit.edu.cn (L.C.); 2 Department of Electronic Engineering, Tsinghua University, Beijing 100084, China

**Keywords:** remote sensing, preprocessing, relative radiation correction, geometric correction, real-time

## Abstract

With the development of remote-sensing technology, optical remote-sensing imagery processing has played an important role in many application fields, such as geological exploration and natural disaster prevention. However, relative radiation correction and geometric correction are key steps in preprocessing because raw image data without preprocessing will cause poor performance during application. Traditionally, remote-sensing data are downlinked to the ground station, preprocessed, and distributed to users. This process generates long delays, which is a major bottleneck in real-time applications for remote-sensing data. Therefore, on-board, real-time image preprocessing is greatly desired. In this paper, a real-time processing architecture for on-board imagery preprocessing is proposed. First, a hierarchical optimization and mapping method is proposed to realize the preprocessing algorithm in a hardware structure, which can effectively reduce the computation burden of on-board processing. Second, a co-processing system using a field-programmable gate array (FPGA) and a digital signal processor (DSP; altogether, FPGA-DSP) based on optimization is designed to realize real-time preprocessing. The experimental results demonstrate the potential application of our system to an on-board processor, for which resources and power consumption are limited.

## 1. Introduction

Remote-sensing techniques are increasingly used in geological exploration, natural disaster prevention, monitoring, etc. [1,2,3,4,5]. They usually require very high-resolution satellite images, which are texturally-rich and may raise the running costs of systems. However, many remote-sensing satellites must rapidly respond to emergencies, such as fires and earthquakes, and quickly return the region of interest (ROI) of the emergency to the ground station [6]. In general processing procedure, the satellite image data are downlinked to the ground station for processing and analysis. The data size of Earth’s observation satellites often exceeds 10 GB. So, the process of data downlink causes a long delay time, and it severely affects rapid response to emergencies [7,8,9]. On-board processing is a way to effectively improve response speed and provide immediate products for rapid decision-making [10,11,12]. After processing and sending the data about which we are most concerned, the amount of data can be reduced several times. Therefore, by processing the data on-board and downlinking the processing results only, the communication bandwidth of downlink can be reduced. At the same time, the data processing flow of the ground station can simultaneously be accelerated and simplified. Consequently, on-board processing can reduce the cost and complexity of ground processing systems and solve the delay problem in image acquisition, analysis, and application. 

The acquired remote-sensing images may contain uneven radiation brightness stripes and deformation areas, due to the defects of the sensors and the relative movement between satellite platforms and the Earth [13,14,15]. Therefore, the acquired raw data from sensors on satellite platforms cannot be used directly. So, image preprocessing is a necessary step to solve such crucial problems. There are several necessary steps for preprocessing within charge coupled device (CCD) camera images, such as relative radiation correction (RRC), geometric correction (GC), and multi-CCD stitching (MCCDS).

Numerous studies have been performed to satisfy the needs of on-board processing. Cong Li et al. [16] introduced a new volume calculation formula and developed a new real-time implementation of a maximum simplex volume algorithm, which is suitable for real-time, on-board processing. Qian Du et al. [8] employed a small portion of pixels in the evaluation of data statistics to accelerate the real-time implementation of detection and classification. This design achieved fast, real-time, on-board processing by reducing computational complexity and simplifying hardware implementation. 

Scholars have also conducted related studies of architecture implementation and efficient algorithm mapping. El-Araby et al. [10] presented a reconfigurable computing real-time cloud detection system for satellite on-board processing. Kalomiros et al. [17] designed a hardware/software field-programmable gate array (FPGA) system for fast image processing, which can be utilized for an extensive range of custom applications. Winfried et al. [18] designed an on-board, bispectral infrared detection system, which is based on the neural network processor NI1000, a digital signal processor (DSP), and a FPGA. The system can perform on-board radiometric correction, geometric correction, and texture extraction. Botella et al. [19] proposed an architecture for a neuromorphic, robust optical flow based on a FPGA, which was applied in a complicated environment. Multi-core processors and graphic processing units (GPUs) for achieving real-time performance of the Harsanyi–Farrand–Chang (HFC) method for a virtual dimensionality (VD) algorithm was proposed for unmixing [20]. Carlos et al. presented the first FPGA design for the HFC-VD algorithm to realize unmixing [21].

The previously mentioned methods—GPU, FPGA, and DSP—are the most common processors for implementing these algorithms in real time. In a ground processing system, a GPU is the popular choice for a preprocessing system. Although a GPU can provide high computing performance, it consumes considerable energy and cannot achieve the radiation tolerance required for an on-board environment. Therefore, a GPU cannot be adapted to an on-board processing system. To satisfy the requirements of on-board processing, this system should be implemented using a FPGA, which has low power consumption and high radiation resistance [22,23,24]. Considering the computational complexity of a preprocessing algorithm, the use of a DSP as a co-processor is common to perform processes that are not computationally demanding and need to be sporadically executed. Although some publications have designed GC systems based on a FPGA, these systems are not suitable for remote-sensing images [25,26,27] or cannot achieve the complete process [28]. To the best of our knowledge, no such hardware systems have been proposed for remote image preprocessing, probably because of the complex computations and data management required. However, such a preprocessing step should be executed on this platform to achieve higher performance.

The process of image preprocessing can be decomposed into two parts. The first step calculates the model parameters. This step processes small amounts of data but involves complex calculations (such as sine and cosine functions), making it suitable for a DSP. The second step uses the model parameters to perform a pixel-by-pixel, gray-scale calculation and obtain the output image. When the pixels are calculated in this step, parallel calculations are appropriate, because the calculation forms of all the pixels are similar. However, due to the irregularity of the image deformation and other issues, there are several problems in the pixel calculation step. First, the calculation of each pixel coordinate requires many parameters and a large amount of hardware computing resources. Some parameters are involved in each pixel coordinate calculation and must be repeatedly calculated many times, thus wasting considerable time. Therefore, it is necessary to optimize the algorithm to improve computational efficiency. Second, due to the irregularity of the image deformation, the input and output data cannot be strictly correlated with each other, which makes it difficult to implement the pipeline process. Therefore, it is necessary to design the methods for reading and storing the data according to the characteristics of the geometric deformation. Third, existing algorithms use floating-point data for calculations. Compared with fixed-point calculations, floating-point calculations require more resources and more time. Because the amount of image data is large, it is very important to design a fixed-point solution to speed up the process.

Therefore, we optimized the design of the preprocessing algorithm regarding these aspects of the hardware implementation. First, a hierarchical decomposition mapping method based on coordinate transformation is proposed, which can effectively reduce the computation burden of on-board processing. Second, according to the characteristics of the data read and write irregularities, a block mapping design is implemented to avoid wasting time when reading and writing data. Finally, we design a fixed-point algorithm for the RRC and pixel resampling parts. The design can reduce resources and ensure accuracy. Using these technologies, an optical image preprocessing system based on FPGA and DSP coprocessors is designed and implemented. Because our system is designed for on-board processing, we chose processors with high radiation tolerance for space environments.

Thus, our contributions can be summarized as follows: first, we proposed a hierarchical optimization and mapping method to realize the preprocessing algorithm in a hardware structure, which can effectively reduce the computation burden of on-board processing. Second, a FPGA-DSP co-processing system based on optimization is designed to realize real-time preprocessing.

The remainder of this paper is structured as follows. The second section describes the preprocessing algorithm. The third section describes a mapping strategy and optimizing method. The fourth section describes the hardware realization and parallel accelerating design. The fifth section presents the experimental results and comparison with related studies. The last section provides conclusions and plans for future research.

## 2. Preprocessing Method

**Figure 1:**
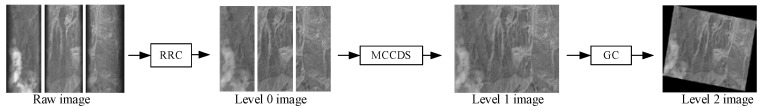
Preprocessing chain. RRC: relative radiation correction; MCCDS: multi-charge-coupled device (CCD) stitching; GC: geometric correction; Level 0: the image after the RRC; Level 1: the image after the MCCDs; and Level 2: the image after the GC.

The complete process for optical remote-sensing CCD image data preprocessing is shown in Figure 1. The process we implemented consists of three parts: RRC, MCCDS, and GC. The input of the preprocessing chain is a raw image with its corresponding ancillary information (imaging time, orbit, attitude, and other necessary information). The output of the preprocessing chain is the georeferenced image. We call the image after the RRC the Level 0 image; the image after the MCCDS is the Level 1 image, and the image after the GC is the Level 2 image.

The RRC is used to remove the systematic noise introduced by the discrepancy in the optical-electronic responses between different detectors and can be described as follows:

(1) $$y_{i}=k_{i}\times x_{i}+b_{i},$$

where *b_i_* and *k_i_* represent the bias and gain coefficients, respectively, of the *i*th detector, which are provided by the manufacturer or calibration laboratory, and *x_i_* and *y_i_* correspond to the digital number value and the at-sensor radiance of the *i*th detector, respectively [29].

The MCCDS is based on the virtual CCD and rational function model (RFM). We summarize the process in two steps. First, the image coordinates of the Level 1 image corresponding to a certain number of points in the Level 0 image are solved using the rigorous imaging model and the orbit, attitude, and auxiliary information. The Level 1 image rational polynomial coefficients (RPCs) for the RFM are calculated based on these coordinate relationships. Second, for each coordinate in the required Level 1 image, the corresponding coordinate in the Level 0 image is calculated via the RPCs, and the gray value is obtained by resampling. The RFM that is employed in this process is expressed as follows:

(2) $$\begin{array}{c} s=(x_{0}+a\times x_{1}+b\times x_{2}+a\times b\times x_{3})\times s_{scale}+s_{off}\\ l=(y_{0}+a\times y_{1}+b\times y_{2}+a\times b\times y_{3})\times l_{scale}+l_{off} \end{array},$$

where *a* and *b* are the row coordinates and column coordinates, respectively, of the Level 1 image; *s* and *l* are the row coordinates and column coordinates, respectively, of the Level 0 image; *x*_0_, *x*_1_, *x*_2_, *x*_3_, *y*_0_, *y*_1_, *y*_2_, and *y*_3_ are the respective polynomial coefficients; *s_scale_* and *l_scale_* are the scale factors; and *s_off_* and *l_off_* are the offsets.

The purpose of the GC is to correct the deformations that occur during imaging [30]. GC methods are divided into parametric and non-parametric models [31]. For on-board processing, it is more suitable to choose the parametric model, because the orbital information of the satellite platform can be obtained. The GC is based on the RFM. We summarize the process in two steps. First, the geographic coordinates in the Level 2 image that correspond to a certain number of points in the Level 1 image are solved using the rigorous imaging model, the RFM of the Level 1 image, and other information. Then, the RPCs for the RFM are solved based on the coordinate relationships. Second, for each geographic coordinate of the requested region in the Level 2 image, the corresponding image coordinate in the Level 1 image is calculated via the RPCs, and the gray value is obtained by resampling. The RFM used in this process is expressed as follows:

(3) $$\begin{array}{c} s=\frac{x_{0}+lon\times x_{1}+lat\times x_{2}+h\times x_{3}}{lon\times x_{4}+lat\times x_{5}+h\times x_{6}+1}\times s_{scale}+s_{off}\\ l=\frac{y_{0}+lon\times y_{1}+lat\times y_{2}+h\times y_{3}}{lon\times y_{4}+lat\times y_{5}+h\times y_{6}+1}\times l_{scale}+l_{off} \end{array},$$

where *s* and *l* are the pixel coordinates of the Level 1 image, *x*_0_–*x*_6_ and *y*_0_–*y*_6_ are RPCs, *lon* is the longitude, *lat* is the latitude, *h* is the elevation, *s_scale_* and *l_scale_* are the scale factors, and *s_off_* and *l_off_* are the offsets.

**Figure 2:**
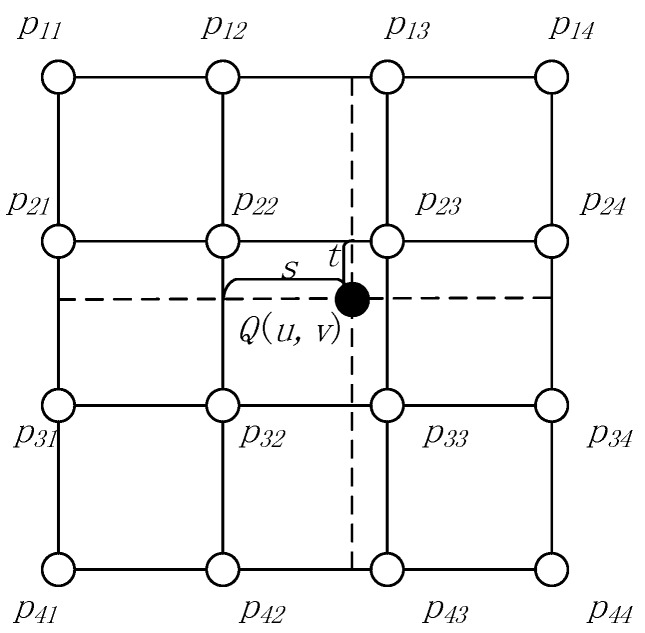
Bi-cubic interpolation method. *Q*(*u,v*) is the output pixel gray value, (*u,v*) is the sample position, *p*_11_ to *p*_44_ are the original sample pixel gray values, t=v−⌊v⌋, and s=u−⌊u⌋.

After the coordinate transformation, we obtain the coordinates (*s* and *l*) of the image pixels. Because the image is a discrete space grid, resampling is required to obtain the image gray values using the interpolation method. Because the bi-cubic interpolation method yields the best performance, we chose this method for our preprocessing algorithm. The bi-cubic interpolation method is shown in Figure 2, which can be described as

(4) $$\begin{array}{cc} Q(u,v) & =[a_{1}p_{11}+a_{2}p_{21}+a_{3}p_{31}+a_{4}p_{41}]\times b_{1}+[a_{1}p_{12}+a_{2}p_{22}+a_{3}p_{32}+a_{4}p_{42}]\times b_{2}\\ & +[a_{1}p_{13}+a_{2}p_{23}+a_{3}p_{33}+a_{4}p_{43}]\times b_{3}+[a_{1}p_{14}+a_{2}p_{24}+a_{3}p_{34}+a_{4}p_{44}]\times b_{4} \end{array},$$

where

(5) $$\begin{array}{cccc} a_{1}=-t+2t^{2}-t^{3} & a_{2}=1-2t^{2}+t^{3} & a_{3}=t+t^{2}-t^{3} & a_{4}=-t^{2}+t^{3}\\ b_{1}=-s+2s^{2}-s^{3} & b_{2}=1-2s^{2}+s^{3} & b_{3}=s+s^{2}-s^{3} & b_{4}=-s^{2}+s^{3} \end{array},$$

and *Q*(*u,v*) is the output pixel gray value, (*u,v*) is the sample position, *p*_11_ to *p*_44_ are the original sample pixel gray values, t=v−⌊v⌋, and s=u−⌊u⌋.

More descriptions of the image preprocessing are provided in [32,33,34].

## 3. Parallel Accelerating Architecture

The preprocessing algorithm can be divided into two parts for hardware processing. The linear part, which contains a large number of rapid but repetitive computations, is the largest computational burden of on-board, real-time implementation. Because the linear part is a per-image pixel operation, communication with a mass storage resource must be considered. The nonlinear part consists of slower and more complex computations that determine the image quality.

### 3.1. Nonlinear Part Mapping Strategy

#### 3.1.1. Hierarchical Decomposition Mapping Strategy

**Figure 3:**
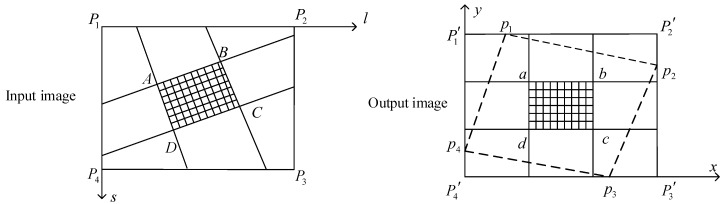
Image block correction method. *P*_1_*P*_2_*P*_3_*P*_4_ is the input image (Level 0 image or Level 1 image), *p*_1_*p*_2_*p*_3_*p*_4_ is the corresponding output image (Level 1 image or Level 2 image), *P*_1_*^’^P*_2_*^’^P*_3_*^’^P*_4_*^’^* is the image range for image storage, *abcd* is one of the image blocks after the output image is divided, and *ABCD* is the corresponding image block in the input image.

The nonlinear parts include the calculation of the RPCs and the coordinates. Although the method in the last section can complete the preprocessing of images, point-by-point calculation renders the hardware system complicated and time-consuming. To satisfy the needs of on-board processing, optimization of the algorithm is important. To balance the accuracy and complexity of the preprocessing algorithm, we split the image during the MCCDS and GC steps, as shown in Figure 3. *P*_1_*P*_2_*P*_3_*P*_4_ is the input image (Level 0 image or Level 1 image), *p*_1_*p*_2_*p*_3_*p*_4_ is the corresponding output image (Level 1 image or Level 2 image), and *P*_1_*^’^P*_2_*^’^P*_3_*^’^P*_4_*^’^* is the image range for image storage. *abcd* is one of the image blocks after the output image is divided, and the corresponding image block in the input image is *ABCD*. For each image block, a corresponding set of RPCs exists. A smaller image block produces more accurate image correction and higher computational complexity. Therefore, the size of the image block is an important parameter.

In image block correction processing, the coordinate calculation in each image block involves many parameters, which enables a reduction in the number of computations.

The RFM in Section 2 can be simplified to the following formula when converting coordinates from a Level 1 image to a Level 0 image:

(6) $$\begin{array}{l} s=s_{0}+(t_{0}+n\times t_{1})\times s_{scale}\\ l=l_{0}+(t_{2}+n\times t_{3})\times l_{scale} \end{array},$$

where *n* is the column number of one block in the Level 1 image. The remaining parameters are expressed as follows:

(7) $$\begin{array}{ll} t_{0}=m\times (s_{1}+s_{4}) & t_{1}=s_{2}+m\times s_{3}+s_{5}\\ t_{2}=m\times (l_{1}+l_{4}) & t_{3}=l_{2}+m\times l_{3}+l_{5} \end{array},$$

where *m* is the row number of one block in the Level 1 image. The remaining parameters are expressed as

(8) $$\begin{array}{ll} s_{0}=(x_{0}+a_{0}\times x_{1}+b_{0}\times x_{2}+a_{0}\times b_{0}\times x_{3})\times s_{scale}+s_{off} & s_{1}=\Delta a\times x_{1}\\ s_{2}=\Delta b\times x_{2}s_{3}=\Delta a\times \Delta b\times x_{3}s_{4}=a_{0}\times \Delta b\times x_{3} & s_{5}=\Delta a\times b_{0}\times x_{3}\\ l_{0}=(y_{0}+a_{0}\times y_{1}+b_{0}\times y_{2}+a_{0}\times b_{0}\times y_{3})\times l_{scale}+l_{off} & l_{1}=\Delta a\times y_{1}\\ l_{2}=\Delta b\times y_{2}l_{3}=\Delta a\times \Delta b\times y_{3}l_{4}=a_{0}\times \Delta b\times y_{3} & l_{5}=\Delta a\times b_{0}\times y_{3} \end{array},$$

where *a*_0_ and *b*_0_ are the initial row number of the block and the initial column number of the block, respectively. Δ*a* and Δ*b* are the row step and column step, respectively; both are set to one in this algorithm. The remaining parameters are described in Section 2.

**Figure 4:**
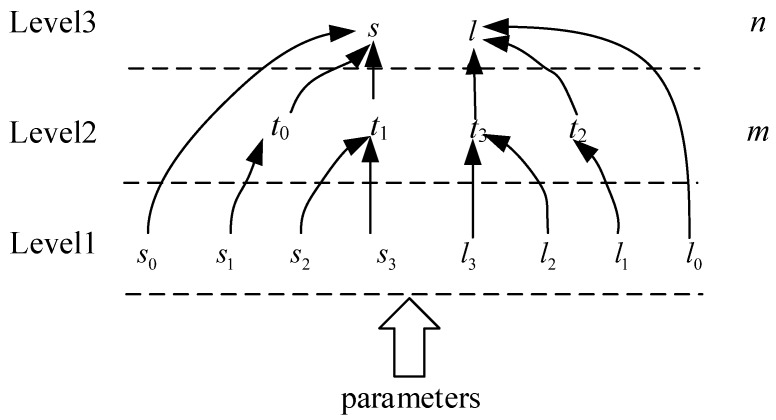
Hierarchical parameters calculation flow of Level 0 to Level 1.

So, we divide these parameter calculations into three levels. The relationships among the different levels are shown in Figure 4. The first-level parameters only need to be calculated once during an image block. The second-level parameters need to be calculated once during each line of a block. The third-level parameters have to be calculated per pixel. 

**Figure 5:**
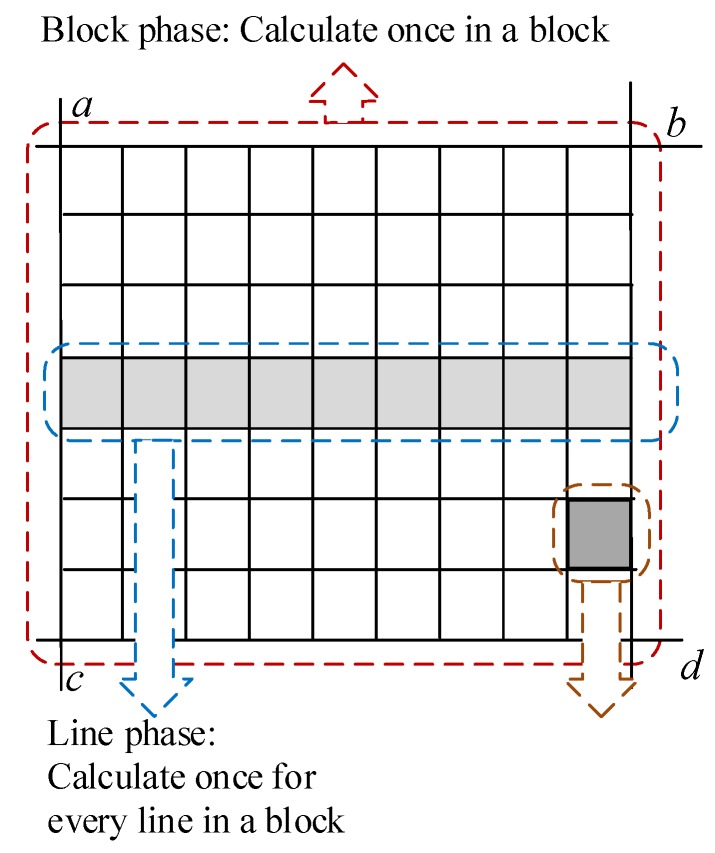
Different calculation phases in an image block.

The three levels are also shown in Figure 5. The block phase, the line phase, and the point phase correspond to Expression (6), Expression (5), and Expression (4), respectively. The block phase is processed only when the initial parameters are provided for each image block. The results calculated by the block phase are sent to the line operation phase. After receiving the block calculation data, each line of the image block is calculated in the line phase, and the results are sent to the point phase. In the point phase, a point-by-point calculation occurs according to the received parameters. By optimizing the process, we can reduce the number of additions and multiplications when performing the point-by-point calculations, which reduces the use of many resources.

The RFM in Section 2 that performs a coordinate transformation from a Level 2 image to a Level 1 image can be simplified. Because *lon* and *lat* are incremented by a fixed-step size and are redundant in the calculations, the process can be transformed into

(9) $$\begin{array}{l} s=\frac{t_{0}+n\times s_{2}+h\times x_{3}}{t_{1}+n\times s_{5}+h\times x_{6}}\times s_{scale}+s_{off}\\ l=\frac{t_{2}+n\times l_{2}+h\times y_{3}}{t_{3}+n\times l_{5}+h\times y_{6}}\times l_{scale}+l_{off} \end{array},$$

where *n* is the number of steps in the longitude in the Level 2 image. The remaining parameters are expressed as

(10) $$\begin{array}{ll} t_{0}=s_{0}+m\times s_{1} & t_{1}=s_{3}+m\times s_{4}\\ t_{2}=l_{0}+m\times l_{1} & t_{3}=l_{3}+m\times l_{4} \end{array},$$

where *m* is the number of steps in the latitude in the Level 2 image. The remaining variables are described in the following formula:

(11) $$\begin{array}{lll} s_{0}=x_{0}+lon_{0}\times x_{1}+lat_{0}\times x_{2} & s_{1}=\Delta lon\times x_{1} & s_{2}=\Delta lat\times x_{2}\\ s_{3}=lon_{0}\times x_{4}+lat_{0}\times x_{5}+1 & s_{4}=\Delta lon\times x_{4} & s_{5}=\Delta lat\times x_{5}\\ l_{0}=y_{0}+lon_{0}\times y_{1}+lat_{0}\times y_{2} & l_{1}=\Delta lon\times y_{1} & l_{2}=\Delta lat\times y_{2}\\ l_{3}=lon_{0}\times y_{4}+lat_{0}\times y_{5}+1 & l_{4}=\Delta lon\times y_{4} & l_{5}=\Delta lat\times y_{5} \end{array},$$

where Δ*lon* and Δ*lat* are the steps in the latitude and the longitude, respectively. For the calculation order of each parameter, we divide the coordinate transformation process into three phases, as shown in Figure 5, with the same order employed for the Level 1 image to the Level 0 image. The block phase, the line phase, and the point phase correspond to Expression (9), Expression (8), and Expression (7), respectively.

**Figure 6:**
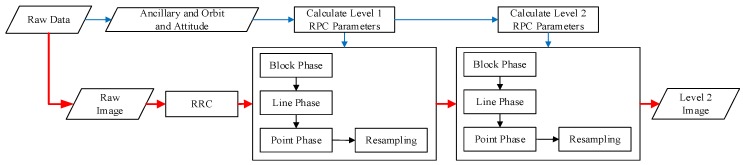
Preprocessing system work flow. The parallelograms represent data, and the rectangles represent the processing phases. The image data flow is shown in solid red lines, and the attitude and ancillary data flows are shown in solid blue lines.

After optimization, we can obtain the new preprocessing chain, as shown in Figure 6, in which the parallelograms represent data and the rectangles represent the processing phases. The image data flow is shown in solid red lines, and the attitude and ancillary data flows are shown in solid blue lines. The data from the camera is separated into the image and the auxiliary data. The auxiliary data are used to calculate the RPCs of the Level 1 image and the Level 2 image. After processing the raw images using the RRC, the images are divided into blocks. Each image block undergoes the block phase, line phase, point phase, and resampling processing based on the respective RPCs. After this processing, we obtain the Level 2 image.

#### 3.1.2. Complexity Analysis

After optimizing the calculation process, the computation times are reduced. For an image of 4096 × 4096 pixels, if we divide it into 32 × 32 blocks (each block is 128 × 128 pixels), then we can eliminate 133,892,672 additions and 166,692,864 multiplications. Detailed information is listed in Table 1.

### 3.2. Linear Part Mapping Strategy

The linear part primarily includes pixel grey calculations and data access. These operations need to calculate the grey values of each block of image. To satisfy the on-board processing needs, we needed to improve the efficiency of data access and calculation.

#### 3.2.1. Data Access Pattern

**Figure 7:**
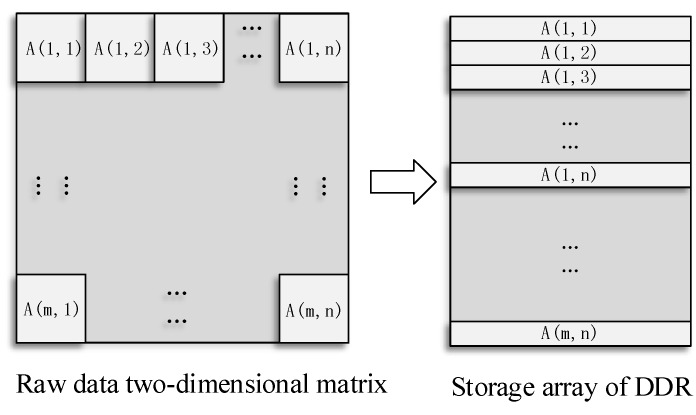
Block mapping method. DDR: dual data rate.

**Figure 8:**
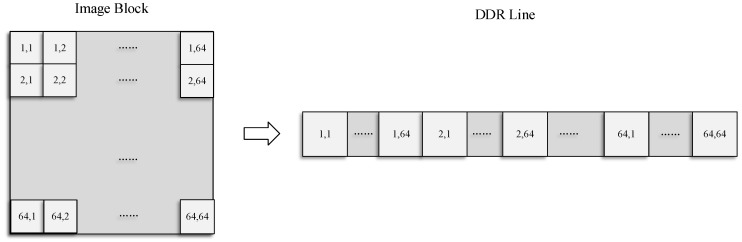
Single image block mapping method.

The principle of the mapping storage method is to optimize and balance the line and block data access rate. The MCCDS and GC must adopt the block correction method. If an image is stored in a normal sequence in the dual data rate (DDR) synchronous dynamic random-access memory (SDRAM), then the read and write processes will involve cross-banking, which lowers the efficiency. We needed to design a high-efficiency pattern based on the row-major format, which is a common method for storing multidimensional arrays. Therefore, we designed a form of mapping memory locations according to the image block, as shown in Figure 7, in which a single image block is mapped, as shown in Figure 8. The image is divided into *m × n* blocks, and each block has 64 × 64 pixels. The image is stored in the DDR with *m × n* rows, and each row stores the data of one image block. In the data read process, reading each image block corresponds to reading a row in the DDR. Thus, we can achieve the maximum efficiency for reading and writing data.

During reading and writing, the decoding must be performed according to the corresponding address. Because each operation is performed according to the image block, the decoding is divided into two steps. First, the image coordinates are mapped to the image block number. Second, the image block number is mapped to the DDR address. The relationship between the image coordinates (*x*, *y*) and the image block coordinates (*m*, *n*) is as follows:

(12) $$\{\begin{array}{c} m=\left\lfloor x/64\right\rfloor \\ n=\left\lfloor y/64\right\rfloor \end{array}.$$

The relationship between the image block coordinates (*m*, *n*) and the row number *s* of a single bank in the DDR is

(13) s=m×N+n.

When external data are written into the DDR, the data controller transforms the image according to the above method. In the subsequent image preprocessing process, the data controller only needs to read and write data using an image block according to the coordinate relationship. The inverse transformation of the image data only has to be done once in the final output process.

#### 3.2.2. Parallel Processing Data Access

To realize parallel processing, we needed to analyze the data processing procedure. The image blocks can be sequentially read and written during the RRC process. When performing the MCCDS and GC processes, it is necessary to calculate the coordinates using the RFM and then perform resampling calculations according to the image coordinates. Due to the irregularity of coordinate transformation, it is difficult to predict the pixel positions required for each participating operation, which will affect the efficiency of the pipeline processing performance. Researchers [35] have proposed a parallel computing strategy that can weaken the influence of the above characteristics, but that strategy is not suitable for implementation with a FPGA. When using a FPGA for data processing, a suitable rule for data reading and storage methods can make the processor perform better. Therefore, we designed rules for data reading and writing for the preprocessing algorithm.

**Figure 9:**
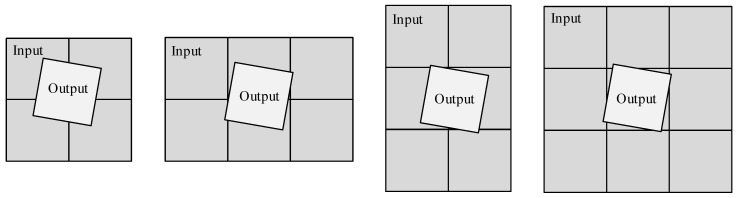
Positional relationship of output and input data.

During the MCCDS and GC steps, the input image grid position corresponding to each output image changes after the grid is divided. Therefore, the amount of data read from the DDR cannot be consistent every time. To solve this problem, we analyzed the positions of the grids. As shown in Figure 9, the output grids are primarily mapped to the input grids in four situations. Therefore, the maximum number of input grids corresponding to each output grid is nine (but not all blocks will be calculated). Therefore, we designed nine random-access memory (RAM) areas for reading data in this module; each area is 1 k × 64 bit and stores 64 × 64 pixels. We also designed two RAM areas for the output data, which can ensure that the pipeline writes the output image block. We designed 16 blocks instead of nine to read the data to ensure that the demand was still met in the event of a large deformation.

**Figure 10:**
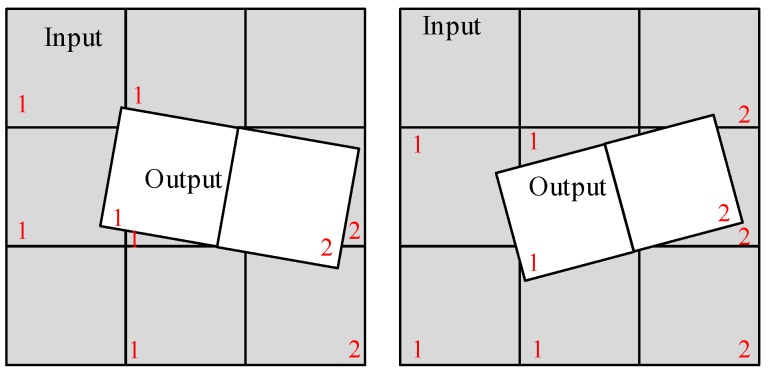
Image block read order. Label 1 in the gray-colored block is the input data that has been read, label 1 in the white-colored block is the currently calculated output image, label 2 in the white-colored block is the next output image block to be calculated, and label 2 in the gray-colored blocks is the data that must be read.

Because coordinate transformations and resampling calculations require more time than the reading and writing of data, we can allow data reading and writing during the calculations to ensure the functioning of the pipeline. When the current computing module uses the RFM for coordinate transformation, the output of the previous sample block is written into the DDR, and the data required for the next sample block is read from the DDR. To reduce the amount of redundant data reads, only 2–3 input data blocks are read at one time. The input data block ranges must cover the output block corner. As shown in Figure 10, we read different data in different situations: label 1 in the gray-colored block is the input data that has been read, label 1 in the white-colored block is the currently calculated output image, and label 2 in the white-colored block is the next output image block to be calculated. The next output block coordinates will decide the next input image blocks, and the data that must be read is represented by label 2 in the gray-colored blocks.

#### 3.2.3. Fixed-Point Calculation Design

When calculating the gray value of the image pixel, both fixed-point and floating-point data formats can be used. The floating-point format has high precision, but is resource-intensive, complex, and slower. The fixed-point format can be performed quickly and requires fewer resources, but the results are less accurate. The optical CCD image pixel gray scale is always 12 bits; therefore, the accuracy of calculation only has to be better than 12 bits. A fixed-point design for the calculation can thus be achieved without damaging effects. By performing fixed-point processing of the data in the calculation process, it is possible to optimize the use of resources and improve the calculation speed while ensuring data accuracy.

The RCC formula is

(14) $$y_{i}=k_{i}*x_{i}+b_{i},$$

where *x_i_* is the original pixel gray with a 12-bit integer and *y_i_* is the corrected pixel gray, which also must be an integer of 12 bits. To ensure a corrected pixel gray accuracy better than one gray level, both *k_i_ × x_i_* and *b_i_* should have accuracies that are better than 0.1 gray level, which is a 4-bit fractional part. Therefore, *b_i_* is 16 bits, the first 12 bits are the integer, and the last 4 bits are the fractional part. Because *k_i_ × x_i_* should have the same accuracy as *b_i_*, *k_i_* is 28 bits, the first 12 bits are the integer, and the last 16 bits are the fractional part.

Because the results of the coordinate transformation in the MCCDS and GC are not an integer, it is necessary to perform a bi-cubic interpolation on the 16 points around the target pixel to obtain the gray value of the required point. Due to the pixel-by-pixel calculation and the large number of computations, a fixed-point design similar to the RRC is used. Table 2 lists the data structure in the fixed-point format that we have employed in this module. Those parameters have been described in Section 2.

## 4. Realization of the FPGA-DSP Accelerating Platform

To test and verify the functionality and performance of the proposed architecture, we developed a prototype system for preprocessing and conducted a parallel processing analysis.

**Figure 11:**
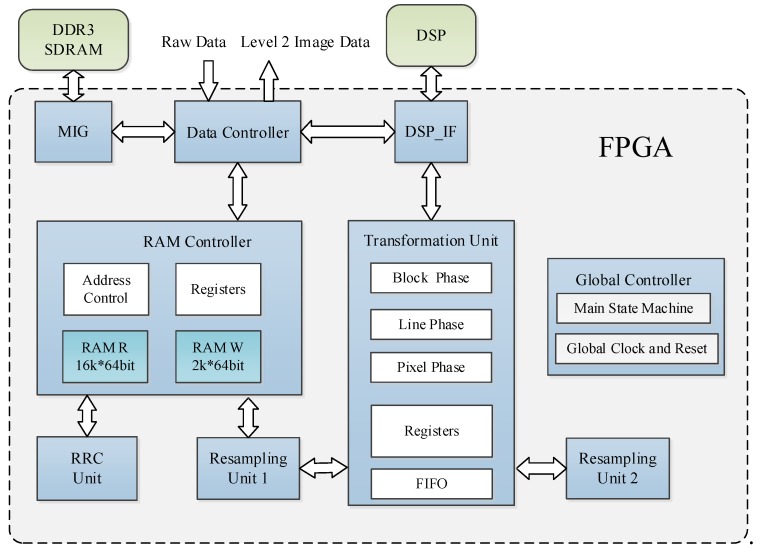
Preprocessing system architecture. FPGA: field-programmable gate array; DSP: digital signal processor; MIG: memory interface generator; RAM: random-access memory; FIFO: first-in-first-out; and SDRAM: synchronous dynamic random-access memory.

This preprocessing system is designed based on a FPGA and a DSP co-processor. The main architectural modules of this preprocessing system are shown in Figure 11. The FPGA receives all the raw data, sends the image to the DDR for storage, and sends the remaining data to the DSP. Then, the FPGA processes the image data, whereas the DSP calculates the parameters of the two RFMs. Because the computations (such as sine and cosine functions) of the RFMs are complicated but utilize few data, the DSP is suitable for this purpose. All image data are processed by the FPGA, which ensures efficient parallelization of the algorithm.

The data controller is responsible for receiving external data and achieving data interactions among the DDR, FPGA, and DSP. The memory interface generator (MIG) is used to control the DDR SDRAM. The RAM controller caches the data that are needed for the RRC unit and resampling unit 1. The RRC unit achieves the RRC process for the entire image. The transformation unit and resampling unit 1 realize the coordinate transformation and resampling processes of the MCCDS and the GC. Resampling unit 2 is applied when a more accurate elevation is required. The DSP_IF unit is used to exchange data between the FPGA and the DSP. We set the FPGA as the main controller in the proposed system. The FPGA will send an interrupt signal to change the work state of the DSP. After receiving the interrupt signal, the DSP will first read the register of the FPGA through external buses. Then, the DSP executes the corresponding process algorithm according to the register value. During this procedure, the DSP reads data from the RAM of the FPGA and then writes the results back to the RAM of the FPGA. When finishing this procedure, the DSP modifies the register value of the FPGA, and the FPGA will perform the specific operation according to the register value, such as reading and writing data from RAM or changing the state machine. The global controller contains the main state machine, which is responsible for the phase transition, global clock, and reset. Global information is propagated to all modules in the form of broadcasts.

The transformation unit performs coordinate transformations based on the RPCs that are sent by the DSP_IF and then sends the coordinate transformation results to resampling unit 1. This module is designed based on the optimization algorithm of Section 3. We designed the block phase, line phase, and point phase in this module. The block phase only needs to be run one time for each image block. The line phase runs once for each line of an image block.

**Figure 12:**
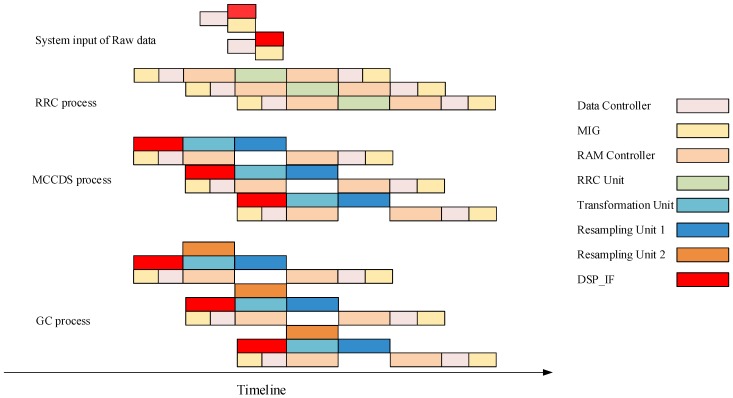
Processing timeline of the system.

The processing timeline is shown in Figure 12, which illustrates the working sequence of the different modules. For each procedure, after sending the data address by the DSP_IF or transformation unit, the data controller and MIG will read or store data for different purposes. Because the speed of reading is substantially higher than the speed of processing, the data controller and MIG consume less time. Because the RAM controller is designed for simultaneously reading and writing data, it can perform different functions during each procedure. As shown in Figure 12, each processing unit (RRC unit, Transformation unit, and Resampling unit) starts working after obtaining data and does not stop until the procedure is ended. All units work on a pipeline and do not waste time waiting for other units.

## 5. Experimental Results

This section uses remote-sensing images to validate the preprocessing system. The verification in this section has two main goals. The first goal is to test and evaluate the effects of the system optimization methods. The second goal is to verify the function of the system and determine whether the system can realize the task of preprocessing. To address an on-board environment, the FPGA in this system was a Xilinx (San Jose, CA, United States) XC6VLX240T, and the DSP was a Texas Instruments (Dallas, TX, United States) TMS320C6701. We mainly used Verilog language to develop the system. In addition, we also used C language and a high-level synthesis tool to develop some computation units, such as the transformation unit and resampling unit. We employed synthetic and real data in our experiments. The synthetic data in this experiment consisted of three CCD push-scan images; the size of each CCD was 12,000 × 30,000 pixels. The real data in this experiment consisted of an image produced by the Gaofen-2 (GF-2) satellite. The image size was 29,200 × 27,620 pixels. 

**Figure 13:**
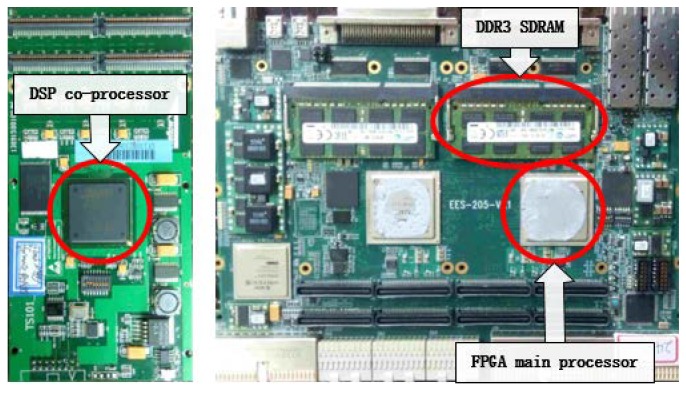
Photo of the hardware system.

A photo of the hardware system that was employed for the preprocessing is shown in Figure 13. In this system, there were two parallel processing units. Each processing unit contained the FPGA and DSP processors and the independent DDR and rapid data transport channel. Thus, we could easily extend the processing ability for different data volumes.

### 5.1. Processing Performance

This section tests the effectiveness of the algorithmic optimization approach that was employed. To evaluate the optimization of the algorithms and structures, we compared the effects of the calculation units (RRC unit, transformation unit, and resampling Unit) before and after optimization.

**Figure 14:**
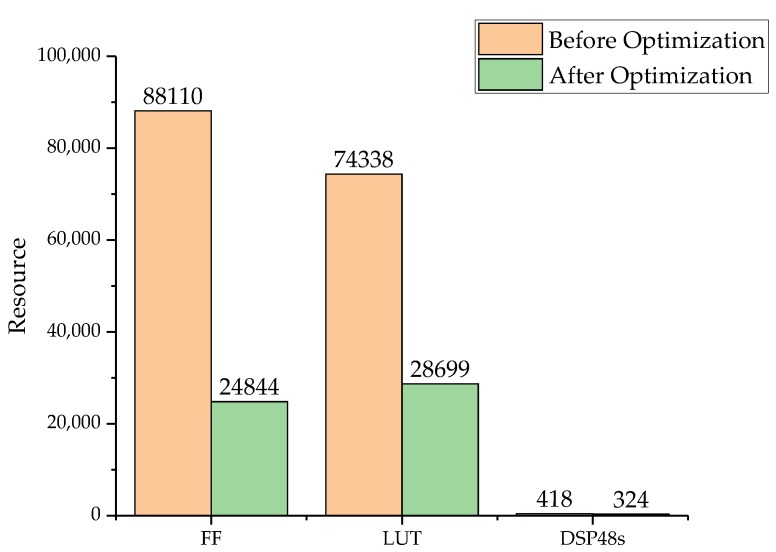
Calculation units’ resource consumption before and after optimization.

To ensure the comparison of identities, we designed the pipeline mode of each unit such that each unit expended the same amount of time for the same image data. The Flip-Flop (FF), Look-Up-Table (LUT), and DSP48 are the most important resources that determine the resource consumption of a FPGA. So, we verified the resource consumption before and after the calculation optimization. The comparison of the resource results is shown in Figure 14. After algorithm optimization and fixed-point calculation design, the consumption of all the calculation resources was lower. Therefore, the design of the hierarchical mapping and fixed-point calculations can reduce the use of resources more than the design with no optimization. Table 3 shows the FPGA resource occupation. The maximal frequency of this design is approximately 163 MHz.

To the best of our knowledge, similar hardware systems for remote image preprocessing have not been proposed; thus, we compare our system with central processing unit (CPU) based and GPU-based systems. The total system processing time for 2.01 GB of data is 11.6 s. For comparison purposes, we also processed 1.12 GB of data and recorded the time. The processing time of each processor in our system (FPGA-DSP co-processor) was compared with the processing times for other systems (CPU and GPU) [36]. Table 4 lists the processing speeds of the different systems. The processing time of an RRC in our system is more than the processing time of a GPU; however, the processing time of a GC is less than the processing time of a GPU. The FPGA design can reach higher speeds, because the FPGA can be more flexible in implementing pipelined and parallel process. Thus, the total processing speed is faster. Due to the relatively slow processing speed of the model parameters calculation by the DSP, the acceleration of the RRC process by increasing the resource usage and waiting for the parameters is unnecessary. Although the system based on a GPU can realize rapid development, it is not suitable for an on-board environment. The power consumption of our system is about 33 W, which includes two pairs of FPGAs and DSPs and the corresponding memory and Input/Output (I/O) devices. In contrast, the power consumption of the traditional GPU-based system is about 200 W. However, NVIDIA has released the embedded GPU, such as Jetson TX2, and the power consumption of an embedded GPU is nearly 8 W per processor. In order to process the same data volume, the power consumption of an embedded GPU system is close to the power consumption of our system. But these embedded GPUs cannot be adapted for an on-board processing system, which needs radiation tolerance. So, our system is more suitable for an energy-constrained and high radiation space environment. Using the FPGA and the DSP enables greater flexibility in configuration and development at higher speeds. Therefore, the advantage of using the FPGA and DSP systems for on-board data preprocessing is irreplaceable.

### 5.2. Real-Time Assessment

To assess the real-time performance, we present the following formula:

(15) $$p=\frac{T_{in}+T_{pro}+T_{out}}{N*T_{in}},$$

where *Tin* and *Tout* represent the time of raw-data input and the processing result output of the processing node, respectively. *Tpro* is the processing delay. *N* is the number of processing nodes. When *p* is less than one, the system can satisfy the real-time requirement. If *p* is larger than one, the system cannot satisfy the real-time requirement. Because one processing node can process an image, the speed of all data processing is positively related to the number of nodes. For the real-time, on-board task, if we only need to obtain a determined area, then one node is sufficient. If we need to process all data that are acquired, two solutions are available. The first solution is to establish additional processing nodes. The second solution is to establish additional memory when the processing time is less than the input time. Then, the system can process the first image when the second image is inputting into the memory. Our system employs the second solution to cope with the low-speed condition. For the GF-2 satellite, the data input time of the 2 GB image data is 1 s. Our system requires 0.89 s to process and output the same data. Thus, our system can satisfy the needs of real-time processing. For actual processing, only part of the image needs to be preprocessed and downlinked. Thus, the processing time will be substantially shorter. Therefore, our system can satisfy the needs of on-board, real-time processing.

### 5.3. Correctness Results

**Figure 15:**
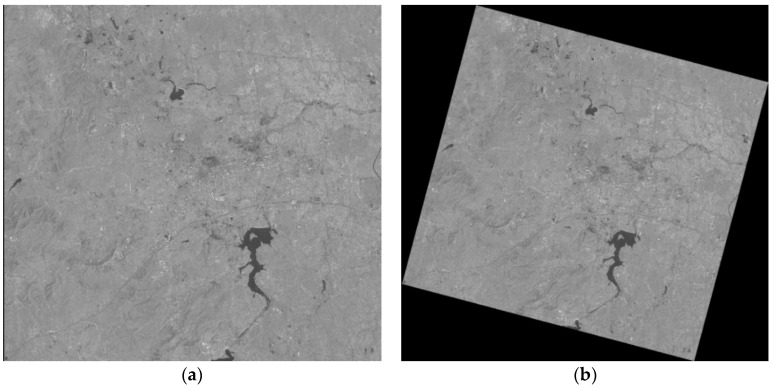
Example of the input and output of the GC processing: (**a**) raw image and (**b**) georeferenced image.

To verify the correctness of our preprocessing system, we compared the results of this system with the results of the personal computer (PC) platform using the root-mean-square error (RMSE) of the output data of the two platforms as the evaluation criteria. The RMSE is expressed as

(16) $$RMSE=\sqrt{\frac{\sum_{i=1}^{w}\sum_{j=1}^{h}{\left(DN_{ij}^{FPGA}-DN_{ij}^{PC}\right)}^{2}}{w*h}},$$

where *ND_ij_^FPGA^* and *DN_ij_^PC^* are the 16 bit integer values of the image pixels that are processed on the on-board platform and PC, respectively. *w* and *h* are the width and height of the Level 2 image. Because the results of the CPU calculation are floating-point data and the results of the FPGA output are fixed-point data, we first compared the RMSE between the output of the FPGA and the floating-point data of the CPU. Then, we compared the RMSE between the FPGA output and the rounding of the CPU output. Table 5 lists the results. As we can see, the maximum RMSE is 0.2934 before the data are rounded. However, after rounding, the RMSE of both becomes zero, which means the corresponding resultant images are perfectly matched. However, the task of image preprocessing needs to obtain only integer-type image data to meet the requirements. Therefore, the fixed-point optimization method adopted by the system satisfies the precision requirements while improving the computational efficiency. Figure 15 provides an example of the input and output of the GC processing.

## 6. Conclusions

This paper presents a FPGA and DSP co-processing system for an optical remote-sensing image preprocessing algorithm. The design can be applied to the rapid responses required for the on-board processing of remote-sensing images. The main contributions of this paper are as follows.

First, we optimized a mapping methodology for the preprocessing algorithm. For the linear part, hierarchical coordinate transformation optimization, a block mapping design, and fixed-point calculation are proposed. The hierarchical optimization can reduce the complexity, the block mapping can prevent the problem of geometric deformation, and the fixed-point design can reduce the time consumption and simplify the design. 

Second, we designed a parallel acceleration architecture for real-time requirements. An optical image preprocessing system that is based on a FPGA and DSP coprocessor was designed and implemented. Because our system is designed for on-board processing, we chose processors with a high radiation tolerance for space environments. The experimental results of this system demonstrate that our system has the potential for application on an on-board processor, for which the resources and power consumption are limited.

Although the current system can achieve the task of preprocessing, it requires the DSP to calculate the RPCs, which limits potential applications. In future research, a preprocessing algorithm based on a full FPGA design will be investigated. By using the FPGA to implement all the processes, the computational efficiency can be further improved and wider applications can also be realized.

## Figures and Tables

**Table 1:** Computation times before and after optimization.

Operation	Additions	Multiplications
*before*	369,098,752	402,653,184
*after*	236,206,080	235,960,320

**Table 2:** Data structures of fixed-point design.

Variable	Sign	Integer	Fractional
*t*, *s*	0	0	32
*t*^2^, *s*^2^	0	0	30
*t*^3^, *s*^3^	0	0	28
*a*_1_, *a*_2_, *a*_3_, *a*_4_	1	0	25
*b*_1_, *b*_2_, *b*_3_, *b*_4_			
*Q*(*u*, *v*)	0	12	0

**Table 3:** FPGA resources occupation (Xilinx xc6vlx240t). LUTs: Look-Up-Table; and FF: Flip-Flop

Parameter	Used	Available
Number of slice registers	41,061	301,440
Number of slice LUTs	39,072	150,720
Number of fully used LUT-FF pairs	20,674	59,459
Number of block RAM/FIFO	230	416
Number of DSP48s	324	768

**Table 4:** Processing times of different systems. CPU: central processing unit; and GPU: graphic processing unit.

Platform Model	CPU (seconds) Intel Xeon E5650 CPU	GPU (seconds) Tesla M2050 GPU	Co-Processor (seconds) XC6VLX240T& TMS320C6701
RRC	3.64	0.23	0.67
MCCDS	-	-	1.67
GC	424.23	8.49	5.40

**Table 5:** Root-mean-square errors (RMSEs) between the CPU processors and the DSP/FPGA co-processors.

Processor	RMSE (before Rounding)	RMSE (after Rounding)
RRC	0.2886	0
MCCDS	0.2934	0
GC	0.2869	0
